# Diagnosis of carotid arterial injury in major trauma using a modification of Memphis criteria

**DOI:** 10.1186/1757-7241-18-61

**Published:** 2010-11-22

**Authors:** Marco Ciapetti, Alessandro Circelli, Giovanni Zagli, Maria Luisa Migliaccio, Rosario Spina, Alessandro Alessi, Manlio Acquafresca, Marco Bartolini, Adriano Peris

**Affiliations:** 1 Anesthesia and Intensive Care Unit of Emergency Department, Careggi Teaching Hospital, Largo Brambilla 3, 50139, Florence, Italy; 2 Resident in Anesthesia and Intensive Care, University of Florence, Largo Brambilla 3, 50139, Florence, Italy; 3 Department of Vascular Surgery, Careggi Teaching Hospital, Largo Brambilla 3, 50139, Florence, Italy; 4 Diagnostic Imaging Department, Careggi Teaching Hospital, Largo Brambilla 3, 50139, Florence, Italy

## Abstract

**Background:**

Incidence of Blunt Cerebrovascular Injuries (BCVI) after head injury has been reported as 0.5-1% of all admissions for blunt trauma, with a high stroke and mortality rate. The purpose of this study is to evaluate if a modification of Memphis criteria could improve the rate of BCVI diagnosis.

**Methods:**

Trauma patients consecutively admitted to Intensive Care Unit (ICU) from Jan 2008 to Oct 2009 were considered for the study. Memphis criteria comprehend: basilar skull fracture with involvement of the carotid canal, cervical spine fracture, neurological exam not explained by brain imaging, Horner's syndrome, LeFort II-III fractures, and neck soft tissue injury. As single criteria modification, we included all patients with petrous bone fracture, even without carotid canal involvement. In all patients at risk of BCVI, 64-slice angio-CT-scans was performed.

**Results:**

During the study period, 266 patients were admitted to the ICU for blunt major trauma. Among them, 162 presented traumatic brain injury or cervical spine fracture. In accordance with the proposed modified-Memphis criteria, 53 patients showed risk factors for BCVI compared to 45 using the original Memphis criteria. Among the 53 patients, 6 resulted as having carotid lesions (2.2% of all blunt major traumas; one patient more than when using Memphis criteria). Anticoagulant therapy with low molecular weight heparin was administered in all patients. No stroke or hemorrhagic complications occurred. Clinical examination at 6-months showed no central neurological deficit.

**Conclusion:**

A modification of a single criteria of Memphis screening protocol might permit the identification of a higher percentage of BCVI. Limited by sample size, this study needs to be validated.

## Introduction

The incidence of Blunt Cerebrovascular Injuries (BCVI) varies from 0.5% to 1% of all admissions for blunt trauma, but this relatively small percentage of patients is affected by a stroke rate ranging from 25% to 58% and a mortality rate ranging from 31% to 59% [1-5].

Although BCVIs are related to severe complications and high mortality rate, controversy exists in literature when defining the patient at risk for these injuries. Four-vessel angiography has been considered the gold standard diagnostic test for the presence of BCVI for a long time. With the increasing availability and accuracy of computed tomography (CT), computed tomography angiography (CTA) has largely supplanted angiography as the primary means of diagnosing BCVI in many institutions, and it has recently been described as a reliable method of screening for the presence of BCVI [3,6-11].

A protocol used for identifying the highest risk patients for BCVI is the one proposed by Miller and co-workers ("Memphis approach") [2]. The aim of the present study was to evaluate if a modification of one of the original Memphis criteria can increase the sensitivity of this screening protocol in BCVI diagnosis,.

## Methods

This study was conducted at the Intensive Care Unit (ICU) of a referral trauma center (Careggi Teaching Hospital, Florence, Italy), and includes trauma patients admitted from January 2008 to October 2009. The ICU database (FileMaker Pro 5.5v2, FileMaker, Inc, USA) was used for data registration and collection. To identify patients at highest risk for BCVI, the Memphis approach [2] was adopted and modified: all petrous bone fractures instead of lesions limited to the involvement of the carotid canal were included (Table 1), as previously suggested as risk factor for carotid arterial injury by Biffl and co-workers [12]. The study was conducted in accordance with the principles of the Declaration of Helsinki and Internal Review Board approved the study.

**Table 1:** Screening protocols for BCVI diagnosis: original Memphis [2] and (*italic *format) modified criteria

Screening protocol criteria
Basilar skull fracture with involvement of the carotid canal
*Basilar skull fracture with involvement of petrous bone*
Cervical spine fracture
Neurological exam not explained by brain imaging
Horner's syndrome
LeFort II or III fracture pattern
Neck soft tissue injury (seatbelt sign or hanging or hematoma)

For each patient, demographic and clinical data were collected: age, Glasgow Coma Scale (GCS), Injury Severity Score (ISS), Revised Trauma Score (RTS), Trauma and Injury Severity Score (TRISS), length of stay (LOS) in ICU and in hospital, intra-ICU and hospital mortality, and neurological deficit at 6-months follow-up. Reports of all the relevant radiographic studies were also examined to determine diagnostic and confirmatory studies, grade of injury, evidence of cerebral or cerebellar infarction, and progression of the injury on follow-up imaging.

At admission, patients were examined in the 8-slice CT of Emergency Department as provided by the institutional protocol for major trauma admission. After radiological identification of the presence of modified Memphis criteria, a 64-slice CTA (64-slice multi-detector, General Electric, Fairfield, Conn.) was performed.

Protocol consists of a timed contrast injection, with images obtained from the aortic arch to the clinoids. Imaging parameters were the following: axial slices were collimated at 2.5 mm with a pitch of 1, but additional data acquisition from multiple slices per rotation allowed reconstruction in the sagittal plane to a resolution of 0.63 mm. Contrast was injected at a rate of 3 mL per second after a 25 second delay for 25 seconds. Physicians involved in the analysis of the CT scans were the radiologist, surgeon, intensivist. Grading of carotid arterial injury was assessed using the scale proposed by Biffl and colleagues (Table 2) [13].

**Table 2:** Biffl Scale for blunt carotid arterial injury [13]

Injury grade	Description
**1**	Luminal irregularity or dissection with < 25% luminal narrowing
**2**	Dissection or intramural hematoma with > = 25% luminal narrowing
**3**	Pseudoaneurysm
**4**	Occlusion
**5**	Transection with free extravasation

## Results

### Overall trauma patients

During the study period, 266 patients were admitted to the ICU for major trauma. Among them, 162 patients (60.9%) had brain injury and/or cervical spine fracture: 32 patients had isolated brain injury, 51 had brain injury and major facial fractures, and 13 patients had brain injury and cervical spine fractures. One patient presented traumatic brain injury, cervical spine fractures and major facial trauma. In 54 cases of head trauma, chest injuries were found. In 11 patients, also abdomen and extremities injuries were present (Table 3).

**Table 3:** Baseline and clinical characteristics of patients admitted for major head trauma during the study period

Number	162
**Age, years**	47 (14-86)
**Injuries, % (N)**	
Brain injury with other thoracic lesions	33.4% (54)
Brain injury and major facial fractures	31.5% (51)
Isolated brain injury	19.6% (32)
Brain injury and cervical spine fractures	8.1% (13)
Brain injury with other lesions (non thoracic)	6.8% (11)
Brain injury and major facial fractures and cervical spine fractures	0.6% (1)
**Pre-hospital GCS (median)**	9 (3-15)
**ISS (median)**	26.9 (15-57)
**RTS (median)**	5.9 (1.8-7.8)
**TRISS (median)**	0.7 (0.1-0.9)
**ICP transducer, % (N)**	36.4% (59)
**Percutaneous tracheotomy, % (N)**	48.1% (78)
**ICU LOS, days**	8 (1-28)
**Hospital LOS, days**	17 (7-45)
**Intra-ICU mortality, % (N)**	19.1% (31)

Continuous variables are expressed as medians with 25th to 75th interquartile range (IQR).

GCS: Glasgow Coma Scale; ISS: Injury Severity Score; RTS: Revised Trauma Score; TRISS: Trauma and Injury Severity Score; ICP: intracranial pressure; LOS: length of stay.

Using the original Memphis approach, 45 patients resulted at risk of BCVI (Table 4), whereas, according to the proposed modified Memphis criteria, 53 patients showed risk factors for extra-cranial cerebrovascular injuries (19.9% of the total of major trauma; 32.7% of traumatic brain injury). All of the patients were screened with CTA within 12 hours after admission to the ICU, and 6 resulted as having lesions of extra-cranial vessels (2.2% of all trauma patients; 11.3% of patient with risk factors) (Table 4).

**Table 4:** Patients at risk of BCVI following the original and the modified Memphis criteria

Injuries	Patients at risk according to Memphis criteria (N)	Patients at risk according to modified Memphis criteria (N)	Patients with BCVI (N)
**Petrous bone fractures**	8	16	1
**LeFort II-III fractures**	14	14	0
**Cervical spine fractures**	13	13	0
**Petrous bone + LeFort II-III fractures**	7	7	3
**Cervical spine + LeFort II-III fractures**	1	1	1
**Cervical spine + petrous bone fracture**	1	1	1
**Neck soft tissue injury**	1	1	0
**Total**	**45**	**53**	**6**

### Patients with BCVI

Patients positive to BCVI screening reported higher trauma scores (ISS, RTS and TRISS), worse on-scene GCS, and longer ICU and hospital LOS than the general population of traumatic brain injury (Table 5). According to the Biffl Scale (Table 2), artery lesions were classified as grade 1 in 2 cases (2 patients), grade 2 (1 patient), grade 3 (2 patients), and grade 4 (1 patient). Of interest, one patient with BCVI would not have been screened if the original Memphis criteria had been used, as the carotid canal was not involved.

**Table 5:** Baseline and clinical characteristics of patients with BCVI

Number	6
**Age, years (median)**	31 (19-44)
**Mechanism of injury:**	
• motorcycle collision	2
• motor vehicle collision	1
• fall	2
• bicycle crash	1
**Pre-hospital GCS (median)**	7 (3-13)
**ISS (median)**	49 (34-57)
**RTS (median)**	4.9 (2.7-7.8)
**TRISS (median)**	0.5 (0.2-0.9)
**ICP transducer, % (N)**	50% (3)
**Percutaneous tracheotomy, % (N)**	100% (6)
**ICU LOS, days (median)**	16 (12-25)
**Hospital LOS, days (median)**	22 (12-50
**Intra-ICU mortality, % (N)**	-

Continuous variables are expressed as medians with 25th to 75th interquartile range (IQR).

GCS: Glasgow Coma Scale; ISS: Injury Severity Score; RTS: Revised Trauma Score; TRISS: Trauma and Injury Severity Score; ICP: intracranial pressure; LOS: length of stay.

### Treatment of BCVI

Dalteparin (150 UI/kg/day) was administered immediately after diagnosis (within 12 hr after ICU admission) in patients without intracranial bleeding, and after 72-96 hr in patients with trauma involving intra-cranial bleeding. No haemorrhage complications were observed. The administration of LMWH continued for the entire treatment in ICU, and the whole period of hospitalization, until the following radiological examination with relative re-evaluation. No neurosurgical or endovascular treatment was performed according to specialist consulting during ICU stay. Stroke did not occur in any of the patients.

### 6-months follow up

At 6-months after ICU discharge, a magnetic resonance angiography of the extra-cranial vessels was performed in all patients, showing no evolution of lesions in two cases, whereas for the other 4 patients, healing was complete. Clinical examination during the 6-months follow up after ICU discharge showed no relevant central neurological deficit.

## Discussion

Despite car safety systems, prevention programs, and pre-hospital/in-hospital strategy improvement of patient care, trauma caused by road accidents is still the major cause of death in the under-40s population [14,15]. The most frequent fatal lesions are brain injury (45.8%), followed by thoracic, abdominal and pelvic trauma (41.6%) [3,4]. The recognition and treatment of BCVI has dramatically evolved over the past two decades. Cerebrovascular injuries have been sporadically described since 1967 through a number of cases [16].

The main finding of our experience consists in the increased sensitivity, even though with he limitation of the sample size, of the Memphis approach by including all patients with petrous bone fracture, independently from the involvement of carotid canal. The percentage of patients screened in our population (19.9%) among those admitted for trauma was higher than previously reported (3.5%-10%) [2,17]. As a difference from original Miller work [2], decision to perform CT angiography was based on CT scan and not on clinical findings. Besides, a single modification of original criteria permitted to extend the screening of the population and the diagnosis and treatment of BCVI in one patient who would not have been considered at risk if the original Memphis criteria were used. Using a modification of the original Memphis criteria, a number of patients underwent to additional radiological examination without any direct benefit: in our opinion, the risk/benefit ratio of X-ray exposure in case of severe trauma can be justified by the possibility to make a potentially lifesaving diagnosis.

Notably, we did not find any vertebral artery injury in our population. This maybe be attributed to the sample size and, consequently, to the limited number of cervical vertebra injuries observed (Table 4). More recently respect to this study period, the importance of petrous bone fracture has been identified in a multivariate logistic regression analysis of a large cohort [18] and underlined in the guideline of the Eastern Association for the Surgery of Trauma [19].

Three basic means of BCVI have been encountered: 1) extreme hyper-extension and rotation; 2) a direct blow to the vessel; 3) vessel laceration by adjacent bone fractures [20]. The most common causes of blunt carotid injury are: 1) hyper-extension of the carotid vessels over the lateral articulation of C1-C3 at the base of the skull; 2) a direct blow to the artery; 3) basilar skull fractures involving the petrous bone or sphenoid portions of the carotid canal. The accepted mechanism for vertebral artery injury results in secondary damage, due to fractures of the transverse foramen through which the vessel courses (C2-C6). Based on the nature of the injury, the traumatic event may cause intimal disruption, pseudo-aneurysm, dissection, and/or thrombosis. Moreover, the lesion can evolve despite a small intimal injury [1].

The overall incidence of BCVI observed in our population (2.2%) is higher if compared to the most recent study on a large trauma population, reporting a BCVI incidence of up to 1% [2,15]. This observation may be related to the use of extended screening together with an improvement of sensibility in diagnosing vessel injury. Nevertheless, the real incidence rate of BCVI could be higher than previously reported. A superior BCVI incidence rate can be found in another recent article by Stein and co-workers [21]. The Authors, analyzing a large population of 12,667 patient in a 30 month period, reported an incidence of BCVI of 2.4% [21]. However, it must be noted that the study of Stein and colleagues was carried out without a defined screening protocol for BCVI, instead being based on physicians' judgment during CT-scan execution.

Literature shows that the most common associated injuries include closed head injuries (50-65%), facial fractures (60%), and thoracic injuries (40-51%). Nearly half of all patients had cervical spine fractures at the time of diagnosis [3,4]. While these data appear different from our sample (Table 3), data shown confirmed that the carotid lesions are often associated to thoracic lesions, and in our case series, half of the patients with BCVI had thoracic region injuries.

The mortality rate of BCVI patients in our experience was 0 compared to the 13% reported in the largest study available [21]. This data is perhaps influenced by the median age which is noticeably lower (31 years old). Another explanation consists in the low-medium grade of lesions according to the grading system described by Biffl and co-workers [13] (Table 2). A timely anti-coagulant treatment was reported to reduce mortality due to the lesions, and prevent blood clots in asymptomatic patients [2]. In our experience, treatment with LMWH was effective in preventing stroke evolution, and none of the patients had bleeding events.

## Conclusions

The early identification of BCVI remains a challenge in trauma patients. However, due to its potentially dramatic consequences, a standardized protocol to guarantee a prompt diagnosis is needed. Here we have shown our experience in which the inclusion of petrous bone fracture might have improved the sensibility of screening criteria. In consideration of the small sample and the single center setting, largest studies are needed to identify a common and shareable screening program based on well defined risk factors.

## Key messages

• Cerebrovascular injuries are rare complication of head and neck trauma but associated with high morbidity and mortality.

• A screening protocol should comprehend whole-body scanning with whole body multidetector computed tomographic scans with contrast media.

• A multi-slice angio-CT-scans should be performed in the presence of risk factors for BCVI.

• The proposed expanded screening protocol (including all patients with petrous bone fractures, no need for carotid canal involvement), needs to be further investigated to confirm its role in increasing the sensitivity in BCVI diagnosis.

## List of abbreviations

BCVI: Blunt Cerebrovascular Injuries; CT: Computed Tomography; CTA: Angio-CT; GCS: Glasgow Coma Scale; ISS: Injury Severity Score; ICU: Intensive Care Unit; LMWH: Low Molecular Weight Heparin; LOS: Lenght Of Stay; RTS: Revised Trauma Score; TRISS: Trauma and Injury Severity Score

## Competing interests

The authors declare that they have no competing interests.

## Authors' contributions

AP, MC, MB and RS designed the study; MC, AC, GZ, MB and AP reviewed the literature; AC collected data; AA performed surgical evaluations; MA and MB examined radiological examinations; MLM performed the follow-up activity; MC, AC, GZ and AP wrote the manuscript. All Authors revised and approved the manuscript.
